# Novel γδ T cell-based prognostic signature to estimate risk and aid therapy in hepatocellular carcinoma

**DOI:** 10.1186/s12885-022-09662-6

**Published:** 2022-06-10

**Authors:** Jingrui Wang, Sunbin Ling, Jie Ni, Yafeng Wan

**Affiliations:** Department of Hepatobiliary and Pancreatic Surgery, Affiliated Hangzhou First People’s Hospital, Zhejiang University School of Medicine, No.261, Huansha Road, Zhejiang, Hangzhou China

**Keywords:** Hepatocellular carcinoma, γδ T cells, Prognosis predition, Tumor immune microenvironment, Tumor mutation burden, RFESD

## Abstract

**Background:**

Numerous studies have revealed that gamma delta (γδ) T cell infiltration plays a crucial regulatory role in hepatocellular carcinoma (HCC) development. Nonetheless, a comprehensive analysis of γδ T cell infiltration in prognosis evaluation and therapeutic prediction remains unclear.

**Methods:**

Multi-omic data on HCC patients were obtained from public databases. The CIBERSORT algorithm was applied to decipher the tumor immune microenvironment (TIME) of HCC. Weighted gene co-expression network analysis (WGCNA) was performed to determine significant modules with γδ T cell-specific genes. Kaplan-Meier survival curves and receiver operating characteristic analyses were used to validate prognostic capability. Additionally, the potential role of RFESD inhibition by si-RFESD in vitro was investigated using EdU and CCK-8 assays.

**Results:**

A total of 16,421 genes from 746 HCC samples (616 cancer and 130 normal) were identified based on three distinct cohorts. Using WGCNA, candidate modules (brown) with 1755 significant corresponding genes were extracted as γδ T cell-specific genes. Next, a novel risk signature consisting of 11 hub genes was constructed using multiple bioinformatic analyses, which presented great prognosis prediction reliability. The risk score exhibited a significant correlation with ICI and chemotherapeutic targets. HCC samples with different risks experienced diverse signalling pathway activities. The possible interaction of risk score with tumor mutation burden (TMB) was further analyzed. Subsequently, the potential functions of the RFESD gene were explored in HCC, and knockdown of RFESD inhibited cell proliferation in HCC cells. Finally, a robust prognostic risk-clinical nomogram was developed and validated to quantify clinical outcomes.

**Conclusions:**

Collectively, comprehensive analyses focusing on γδ T cell patterns will provide insights into prognosis prediction, the mechanisms of immune infiltration, and advanced therapy strategies in HCC.

**Supplementary Information:**

The online version contains supplementary material available at 10.1186/s12885-022-09662-6.

## Background

Primary liver cancer, is one of the most aggressive and common malignant tumors, and has resulted in thousands of cancer-related deaths worldwide [1–3]. Based on traditional pathological classification, hepatocellular carcinoma (HCC) accounts for approximately 80% of all liver cancer cases [2]. Many well-known risk factors for HCC, including aflatoxin exposure, hepatitis virus infection, heavy alcohol intake, type 2 diabetes mellitus, and obesity, play pivotal roles in hepatocarcinogenesis [3, 4]. Because of the extremely complex molecular diversity of genetic and genomic alterations, HCC is considered a highly heterogeneous disease at both the inter- and intratumoral levels [5–9]. Considering the high heterogeneity and diverse etiologies among distinct populations, tumor-node-metastasis (TNM) staging stratification has been ineffective in the prediction of clinical outcomes in patients with HCC [10–13]. Therefore, it is important to develop robust and novel tools for prognostic prediction and therapeutic evaluation to further contribute to the determination of the optimal treatment.

In the past decades, the progression of anti-tumor treatments in immune checkpoint blockade (ICB) immunotherapy, such as anti-cytotoxic T-lymphocyte antigen (CTLA-4) and programmed death (PD)-ligand 1 (L1), have exhibited encouraging therapeutic outcomes in various human malignant tumors [14–17]. According to clinical trial data, only a minor proportion of HCC patients showed an objective response to immunotherapy, indicating the need for further exploration of immunotherapy in HCC [18]. An increasing number of studies have highlighted that the interactions of infiltrating immune cells with tumor components act as key driving factors for tumor progression and therapeutic sensitivity [19–21]. An independent study highlighted that CD4+ T cell exhaustion leads to the acceleration of tumors in HCC [22]. CD8+ T cells produce lymphotoxin-α and lymphotoxin-β, which serve as key promoters of HCC development [23]. Gamma delta (γδ) T cells, a small population of peripheral blood T lymphocytes, express heterodimeric receptors composed of γ and δ chains on the cell surface. Several studies have indicated that γδ T cells with protumor activity serve as pivotal players in cancer development and anti-tumor responses [24, 25]. Therefore, the most promising and robust strategy for comprehensive estimation of tumor sensitivity to therapy may be derived from γδ T cell patterns and classifying HCC patients using specific molecular signatures based on γδ T cell profiling, thereby optimizing therapeutic programs to increase overall survival (OS) probability. However, studies focusing on the prognostic value of γδ T cell patterns in HCC are lacking.

In this study, three HCC sample datasets, GSE54236, The Cancer Genome Atlas-Liver Hepatocellular Carcinoma (TCGA-LIHC), and International Cancer Genome Consortium (ICGC)-FR, were amalgamated to reveal the possible functions of γδ T cell patterns. γδ T cell profiling was performed using the CIBERSORT algorithm, followed by weighted gene co-expression network analysis (WGCNA) to identify the γδ T cell-specific module with the corresponding genes. Candidate genes from the significant module were screened using multiple Cox regression analysis to identify the final 11 key genes. Next, a novel multi-gene prognostic signature and integrated clinical nomogram were constructed. In addition, the possible functions of risk signature in immune infiltration and therapeutic prediction were investigated. Finally, the potential role of RFESD in prognosis prediction, immune infiltration, and cell proliferation in HCC was further investigated. Collectively, the γδ T cell-based risk nomogram was constructed to serve as a reliable predictive indicator and robust prognostic biomarker for clinical outcome prediction, thereby providing direction for clinical therapeutic strategies for HCC.

## Materials and methods

### Collection of multi-omics data

The preparation and downloading of multi-omics data were implemented as described previously [12]. Sequencing profiles for HCC and normal tissue samples were obtained from TCGA-LIHC, ICGC-LICA-FR, ICGC-LIRI-JP, and GSE54236 datasets. The corresponding clinical profiles were also downloaded from TCGA portal, as described previously. All included patients were diagnosed with HCC and had complete mRNA expression values. The gene expression profiles in the fragments per kilobase per million format were obtained from TCGA portal and the ICGC dataset and then transformed into transcripts per kilobase million. The series matrix file of the GSE54236 dataset in quantile-normalized log2 signal intensity was downloaded from the Gene Expression Omnibus (GEO) database. A total of 616 HCC samples (161 from the ICGC-LICA-FR, 374 from TCGA-LIHC, and 81 from the GSE54236 datasets) were collected for subsequent analysis. The R packages “limma” and “sav” were utilized to perform batch calibration and normalize the expression values among the three platforms. Principal component analysis (PCA) was used to validate the normalized results. Next, four categories of somatic mutation data of HCC patients were obtained from TCGA portal. We singled out the mutation files that were obtained through the “SomaticSniper variant aggregation and masking” platform for subsequent analysis. All data were publicly available and had open access; therefore, it was not necessary to obtain ethics committee approval. Data were processed in accordance with the National Institutes of Health TCGA human subject protection (http://cancergenome.nih.gov/publications/publicationguidelines) and related data access policies.

### Landscape of infiltrating immune cells

In total, 616 HCC specimens were subjected to immune infiltration analysis from the HCC samples. Using the CIBERSORT algorithm (http://cibersort.stanford.edu/), the sequencing data of the samples were analyzed and calculated to determine the abundance of 22 tumor-infiltrating immune cell (TIC) subtypes, which represent the cellular constituents of the TIME [26]. After removing the CIBERSORT algorithm results with *p <* 0.05, 125 CIBERSORT results were used for further analysis.

### Weighted gene co-expression network analysis

The sequencing data of the 16,421 genes of HCC patients were used to generate a weighted co-expression network using the WGCNA method. Correlations between sample traits and candidate modules were computed to determine the models highly correlated with traits, in which the genes were further analyzed to screen hub genes [27]. In the current study, we used the immune-infiltrating cell profile, namely the CIBERSORT results, as sample phenotypes and then selected an appropriate soft threshold power (β) value to generate a scaleless network (index of scale-free topologies = 0.90). Then, similar genes were introduced into the same candidate module using the dynamic tree-cutting algorithm with a minimum size of 60. Correlation analysis between module characteristic genes and sample traits was performed using Pearson’s correlation test (*p < 0.05). Finally, we focused on the γδ T cell populations and the module most significantly correlated with γδ T cells was extracted for subsequent analysis.

### Functional enrichment analysis

Pathway enrichment analysis was performed as described previously [21]. Using the R package “org. Hs.eg.db” the Entrez Gene ID for each γδ T cell-related gene was obtained. To elucidate underlying mechanisms of the hub genes related to γδ T cells in biological processes, we implemented the Kyoto Encyclopedia of Genes and Genomes (KEGG) and Gene Ontology (GO) pathways annotation with the “clusterProfiler,” “enrichplot,” and “ggplot2” R packages and visualized the results.

### Construction of γδ T cell-related prognostic signatures

To explore the prognostic role of γδ T cell-associated genes, genes from the most significant module were used to assemble a prognostic risk signature for HCC. A total of 370 HCC samples from TCGA-LIHC project, with a definite report of survival status and duration data, were enrolled for further prognostic signature establishment. The “limma” R package with a false discovery rate < 0.05 and|log2 fold change| > 1 was utilized to recognize differentially expressed genes (DEGs) between normal and HCC samples. DEGs that met the selection criteria were extracted for further analyses. Altogether 370 HCC cases were stochastically assigned into the training and validation sets at a rate of 3:2 for systematic analysis using the project “caret” R package. Both training and validation sets had to comply with the following requirements: [1] cases were stochastically allocated to the training and validation groups and [2] samples in both groups had similar clinicopathological characteristics. Importantly, there were no statistically significant clinical differences between the two sets (Table S5). DEGs that were significantly correlated with OS (*P <* 0.05) were identified using univariate Cox regression analysis in the training set. Next, the least absolute shrinkage and selection operator (LASSO) algorithm using the “glment” R package was analyzed. Subsequently, a multivariate Cox regression model was used to identify hub genes and compute their corresponding coefficients. Finally, a prognostic risk model including five hub γδ T cell-correlated genes was developed, and the risk score was calculated using the following formula:

$$Risk\;score\;=\:\beta\;gene\;1\:\times\:expression\;level\;of\;gene\;1\;+\:\beta\;gene\;2\:\times\:expression\;level\;of\;gene\;2\:+\cdots\;+\;\beta\;gene\;n\;\times\;expression\;level\;of\;gene\;n$$

Here, β is the regression coefficient in the multivariate Cox regression analysis, as described previously [28].

### Validation of prognostic γδ T cell-related signature

The prognostic γδ T cell-related signature was constructed as described previously [12]. HCC samples (*n* = 224) in the training group were stratified into low- and high-risk subgroups by setting the median risk score as the cutoff point. First, the Kaplan-Meier survival curve was plotted using the “survival” R package to identify the prognosis differences. Time-dependent receiver operating characteristic (ROC) curves were analyzed to validate the prognostic values. Univariate and multivariate Cox regression analyses were performed to validate the risk signature as an independent prognostic factor. The predictive precision of the as-constructed risk-score model was further confirmed in the validation group (*n* = 146). To visualize the correlation of risk score with clinicopathological variables, the “pheatmap” R package was used to compare the clinical characteristics of low- and high-risk patients. The ICGC-LIRI-JP cohort with 231 HCC patients was used as an independent testing group and partitioned into high- and low-risk subgroups according to the median threshold of TCGA dataset. The prognosis prediction precision was further validated in the external testing group.

### Correlation of risk score with TIME characterization

Immune infiltration was analyzed as described previously [21]. To uncover the correlation between risk score and tumor-infiltrating immune cells, we implemented seven methods, including the XCELL, TIMER, QUANTISEQ, MCPcounter, EPIC, CIBERSORT, and CIBERSORT-ABS algorithms, to evaluate immune infiltration. Spearman correlation was analyzed to explore the relationship between risk score and immune infiltration status. We compared the differences in the immune-infiltrating cell fractions between the low-and high-risk subgroups.

### Role of risk score in immune checkpoint blockade treatment

According to previous studies, the expression patterns of ICB-related hub targets may contribute to the efficacy of immunotherapy administration [29]. In this study, we identified six hub genes of ICB therapy: PD-L1 (also known as CD274), PD-1 (also known as PDCD1), PD-L2 (also known as PDCD1LG2), CTLA-4, T-cell immunoglobulin domain and mucin domain-containing molecule-3 (also known as HAVCR2), and indoleamine 2,3-dioxygenase 1 (IDO1) in HCC [30–32]. To further explore the potential role of the risk signatures in ICB immunotherapy, the correlation of the prognostic signature with the expression values of six ICB hub genes was analyzed.

### Gene set variation analysis

Gene Set Variation Analysis (GSVA) [33] with the “GSVA” R package was used to explore variations in biological processes between the distinct risk subgroups. Well-defined biological signatures were derived from the gene sets of “c2.cp.kegg.v7.4. symbols.gmt” and “h.all.v7.4. symbols.gmt” (downloaded from the Molecular Signatures Database).

### Process of epigenetic mutation data

The corresponding somatic alteration information of TCGA-LIHC cohort was obtained from TCGA dataset. TMB was defined as the number of somatic, coding, base replacement, and insert-deletion mutations per megabase of the genome examined using non-synonymous and code-shifting indels (insertions and deletions) under a 5% detection limit. The “maftools” R package [33] was employed to detect the number of somatic non-synonymous point mutations within each sample. Somatic alterations in HCC driver genes were observed in samples with low- and high-risk scores.

### Establishment and verification of nomogram

ROC analysis was performed to identify the optimal prognostic indicator, risk score, age, sex, tumor grade, and clinicopathological stage for 1-, 2-, and 3-year OS [34]. To develop a quantitative prognostic tool for HCC patients, a nomogram plot integrating risk score and other clinicopathological features was constructed to predict the 1-, 2-, and 3-year OS rates. A total of 235 HCC samples with complete clinicopathological information (survival status, survival time, tumor grade, clinical staging, and TNM status) from TCGA-LIHC project were used to develop the nomogram. We then plotted the calibration curve to validate the prognostic validity of the nomogram. Finally, the prognosis prediction reliability of the nomogram was validated using the “predict” function of the R package “rms” in the ICGC-LIRI-JP cohort.

### Experimental validation

WRL68 (human pancreatic cell line) and four human pancreatic cancer cell lines (MHCC-97H and HCC-LM3 cells) were purchased from the Cell Bank of the Type Culture Collection of the Chinese Academy of Sciences, Shanghai Institute of Biochemistry and Cell Biology. The cell lines were all cultured in Dulbecco’s minimum essential media (DMEM) plus 10% fetal bovine serum (FBS; Invitrogen, Carlsbad, CA, USA). All cell lines were grown without antibiotics in a humidified atmosphere containing 5% CO_2_ and 99% relative humidity at 37 °C. Three different cell lines were subjected to quantitative real-time polymerase chain reaction (qRT-PCR), which was performed as described previously [4]. All samples were analyzed in triplicate. Glyceraldehyde-3-phosphate dehydrogenase (GAPDH) levels were used as the endogenous control, and the relative expression of RFESD was calculated using the 2-ΔΔCt method. The sequences of the primers used for PCR were as follows:

- RFESD, 5-TGATGGACGACCGTGTATAGTTTGC-3 (forward) and 5-TTATTCCTTTGGAGCACCACTTGGG-3 (reverse);
- GAPDH, 5-CAGGAGGCATTGCTGATGAT-3 (forward) and 5-GAAGGCTGGGGCTCATTT-3 (reverse).

### siRNA interference assay

The small interfering (si) RNAs designed to specifically silence RFESD were purchased from Qingke (Beijing, China). Scrambled siRNA served as the control. The siRNA sequence was as follows:

- si-RFESD: CACUGGAGACUUCAAAGUAAU.
- siRNA was transfected into HCC cells using Lipo8000™ Transfection Reagent (Beyotime, Shanghai, China). Total RNA was isolated 48 h after transfection and assessed by qRT-PCR.

### Cell proliferation assay

For the CCK-8 assay, MHCC-97H cells were plated at 2 × 10^3^ cells per well in 96-well plates and incubated overnight in DMEM supplemented with 10% FBS. The cell proliferation index was measured using a Cell Counting Kit-8 (Beyotime, Shanghai, China) at 0, 24, 48, and 72 h post-transfection according to the manufacturer’s instructions. The absorbance was measured at a wavelength of 450 nm.

For the EdU assay, MHCC-97H cells were seeded into 48-well plates (Corning). When the confluence of MHCC-97H cells reached 80%, BeyoClick™ EdU-488 (Beyotime, Shanghai, China) was used to determine the proliferation rate of the cells. After staining, the cells were imaged using a confocal laser-scanning microscope (ZEISS, Germany).

### Statistical analysis

The Wilcoxon test was used to compare two groups, whereas the Kruskal-Wallis test was used to compare more than two groups. Survival curves were analyzed using the Kaplan-Meier log rank test. The chi-square test was performed to correlate the risk score subgroups with somatic mutation frequency, and Spearman analysis was used to compute the correlation coefficient. The CIBERSORT algorithm results with *p <* 0.05 were included in further analyses. Statistical significance was defined as a two-tailed *p*-value < 0.05.

## Results

### Eliminating of batch effect

**Fig. 1:**
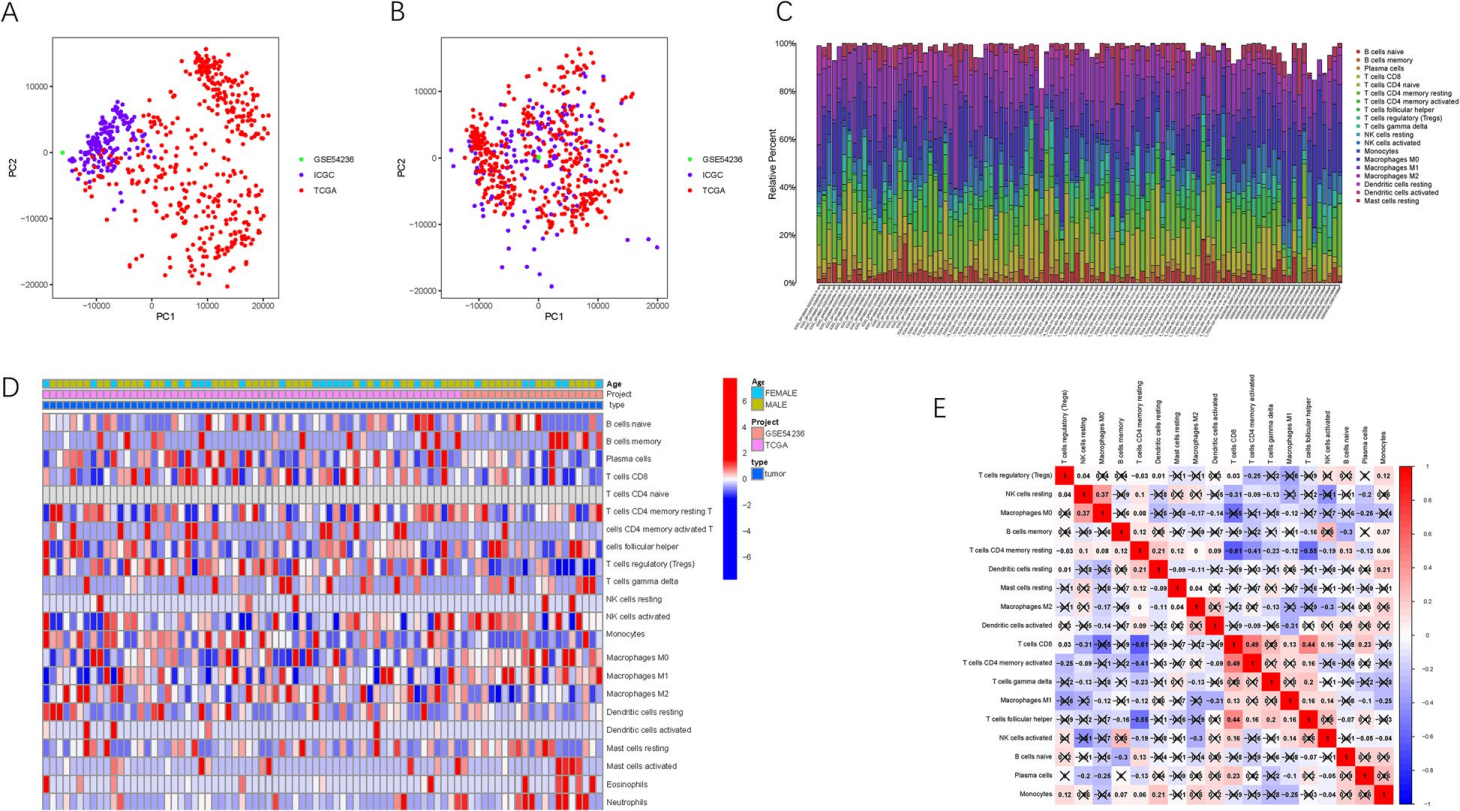
Principal component analysis (PCA) of the gene expression datasets. The points of the scatter plots visualize the samples based on the top two principal components (PC1 and PC2) of gene expression profiles without (**A**) and with (**B**) the removal of batch effect. The colors represent samples from three different datasets, respectively. Landscape of immune cell infiltration in tumor immune environment of HCC. Subpopulation of 22 immune cell subtypes (**C**) and proportional heatmap of the 22 TICs in each HCC samples (**D**). (**E**) Intrinsic correlation of 22 infiltrating immune cells in HCC

To remove the batch effect among the three distinct datasets, “limma” and “sav” algorithms (see Section Method) were performed. In total, 16,421 genes were extracted from the three HCC cohorts. Considering the batch effect, HCC samples were gathered in batches according to the top two principal components of the non-normalized mRNA sequencing (Fig. 1A). After removal of the batch effect, the scatter plot based on the PCA of the normalized expression matrix indicated successful removal of the batch effect by cross-platform normalization (Fig. 1B).

### Landscape of tumor immune microenvironment

The CIBERSORT algorithm was used to determine the comprehensive TIME context (Table S1). Figure 1C shows the relative abundance of the 22 TIC subpopulations. The correlations between TIME patterns and clinical traits were investigated and are presented in a comprehensive heatmap (Fig. 1D). To further uncover the potential intrinsic interactions of infiltrating immune cells, correlation analysis was conducted to visualize the comprehensive landscape of the TIME (Fig. 1E). Notably, CD8+ T cells were the most negatively correlated with resting CD4+ T memory cells, whereas CD8+ T cells were the most positively correlated with activated CD4+ T memory cells.

### Construction of WGCNA co-expression network

**Fig. 2:**
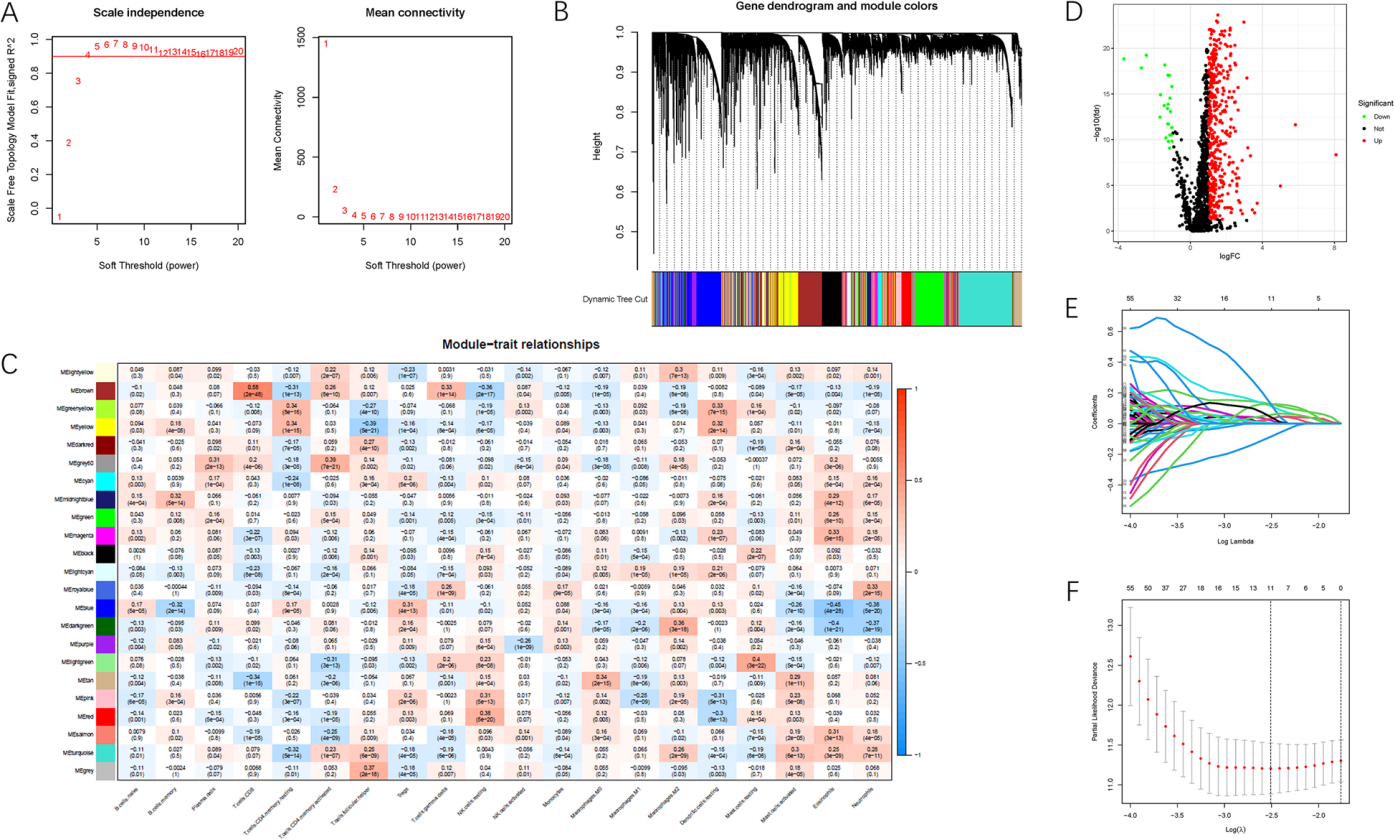
Selection of the appropriate soft threshold (power) and construction of the hierarchical clustering tree. **A** Selection of the soft threshold made the index of scale-free topologies reach 0.90 and analysis of the average connectivity of 1–20 soft threshold power. **B** γδT cells-related genes with similar expression patterns were merged into the same module using a dynamic tree-cutting algorithm, creating a hierarchical clustering tree. **C** Heatmap of the correlations between the modules and immune-infiltrating cells (traits). Within every square, the number on the top refers to the coefficient between the cell infiltrating level and corresponding module, and the bottom is the *P* value. **D** Volcano plot was delineated to visualize the DEGs. Red represented upregulated and green represented downregulated. **E** LASSO coefficient profiles of 440 candidate genes. A vertical line is drawn at the value chosen by 10-fold cross-validation. **F** Ten-time cross-validation for tuning parameter selection in the lasso regression. The vertical lines are plotted based on the optimal data according to the minimum criteria and 1-standard error criterion. The left vertical line represents the 11 genes finally identified

A gene matrix within 15,420 genes and relative subpopulations of 22 immune infiltrations were included for further analyses of the WGCNA co-expression network. The first power value [4] when the index of scale-free topologies achieved 0.90 was set as the optimal soft threshold power (β) to construct the scaleless network (Fig. 2A). Similar expressed genes were assigned to the same candidate module using a dynamic tree-cutting algorithm (module size =60), thereby creating a hierarchical 23-module clustering tree (Fig. 2B). The columns represent 22 TIC abundances, whereas the rows represent the 23 modules with different traits (Fig. 2C). Notably, the brown module was significantly and positively correlated with γδ T cell infiltration (cor = 0.33, *P* = 1e-14). Our primary concern was the infiltration of γδ T cells; therefore, the 1755 genes (Table S2) from the brown module were included in the subsequent analysis.

### GO and KEGG functional annotation

To further reveal the potential role of γδ T cell-specific genes in biological processes, GO and KEGG pathway enrichment analyses were performed (Tables S3 and S4, respectively). The GO pathway enrichment analysis indicated that γδ T cell-specific genes were predominantly involved in T cell activation, regulation of lymphocyte activation, and regulation of immune effector processes in biological processes, and concentrated in the secretory granule membrane, external side of the plasma membrane, and endosome membrane in cellular components. These genes are also involved in cytokine receptor binding, carbohydrate binding, and cytokine activity in molecular function (Fig. S1A-C). As for the KEGG terms analysis, the top three enriched pathways were cytokine-cytokine receptor interaction, Epstein-Barr virus infection, and tuberculosis (Fig. S1D).

### Construction of prognostic risk signature

In total, 448 γδ T cell-related hub genes (23 downregulated genes and 425 upregulated genes) were identified as DEGs between tumor and normal tissue samples (Fig. 2D). To explore the prognosis prediction capability of these genes, expression data and clinical data were extracted from TCGA-LIHC project data. Using univariate Cox regression, 194 γδ T cell-specific genes were screened for prognostic significance (*P <* 0.05, Table S6). Next, the LASSO algorithm was performed to identify 11 hub γδ T cell-related genes (Fig. 2E, F). Using a multivariate proportional hazards model, we identified five γδ T cell-specific genes (ATP1B3, PZP, ST6GALNAC4, RFESD, and IFRD2) as the final hub genes, among which PZP was indicated as a favorable prognostic indicator (all hazard ratios [HRs] < 1), whereas the other four genes (ATP1B3, ST6GALNAC4, RFESD, and IFRD2) were considered risk prognostic factors (all HRs > 1, Table S7). Gene expression values based on TCGA database showed that the expression levels of most genes were significantly and abnormally regulated in HCC samples relative to normal tissues (Fig. S2A-E). In addition, survival analysis between the low- and high-gene expression subgroups showed that abnormal mRNA expression of most hub genes resulted in significantly distinct prognostic outcomes (most *P* < 0.05, Fig. S3A-E).

Finally, these five hub genes were introduced into the prognostic risk model for HCC. The risk score was calculated as follows:

$$Risk\;score\:=\:(0.3115\:\times\;expression\;level\;of\;ATP1B3)\:+\:(0.3777\:\times\:expression\;level\;of\;ST6GALNAC4)\:+\:(0.9482\:\times\:expression\;level\;of\;RFESD)\:+\:(0.3308\:\times\:expression\;level\;of\;IFRD2)\;-\;(0.5348\:\times\:expression\;level\;of\;PZP)$$

Finally, each HCC sample, together with the corresponding risk score, was stratified into low- and high-risk groups according to the median cutoff value.

### Validation of prognostic risk signature

**Fig. 3:**
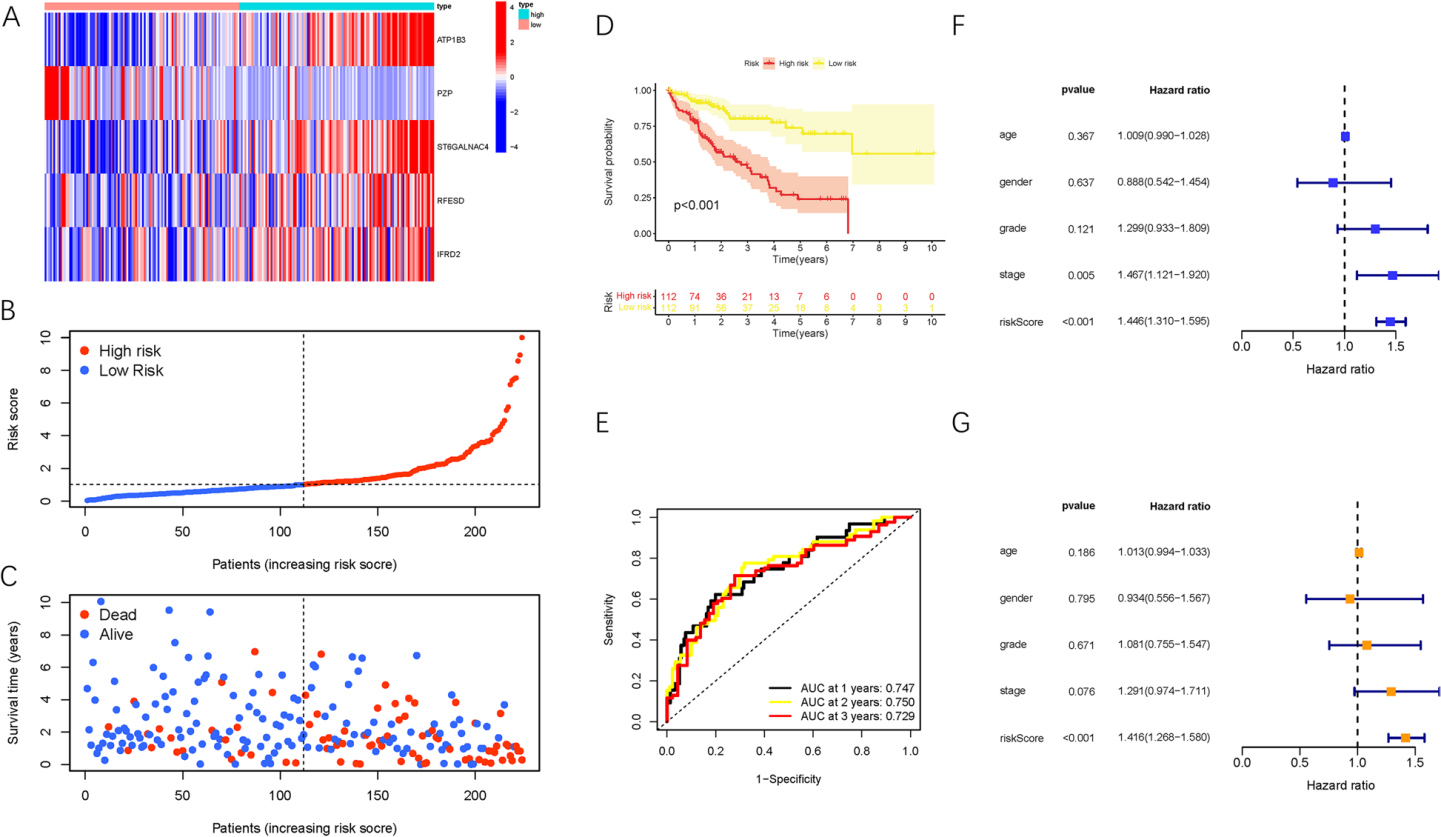
Establishment of the prognostic risk signature. **A** Heatmap presents the expression pattern of 5 hub genes in each patient, where the colors of red to blue represented alterations from high expression to low expression. **B** Distribution of multi-genes model risk score. **C** The survival status and duration of HCC patients. **D** Kaplan–Meier curve analysis presenting difference of overall survival between the high-risk and low-risk groups. **E** ROC analysis of the risk scores for overall prognosis prediction. The AUC was calculated for ROC curves, and sensitivity and specificity were calculated to assess score performance. **F** Univariate Cox regression results of overall survival. **G** Multivariate Cox regression results of overall survival

Figure 3A shows the distributions of the hub gene expression values in the patient and risk groups. In addition, risk score allocations and dot pot of survival status highlighted that high-risk HCC patients exhibited shorter OS times (Figure 3B, C). Kaplan-Meier survival analysis revealed that high-risk patients showed significantly poorer prognosis relative to low-risk patients (*P* < 0.001, Fig. 3D). Moreover, the area under the curve (AUC) was 0.747, 0.750, and 0.729 at 1, 2, and 3 years, respectively (Fig. 3E). Next, the univariate Cox analysis determined the HR for the risk score was 1.446 (95% confidence interval [CI]:1.310–1.595; Fig. 3F). Furthermore, multivariate Cox regression analysis (HR = 1.416, 95% CI:1.268–1.580; Fig. 3G) demonstrated that risk score is an independent prognosis prediction factor in HCC.

**Fig. 4:**
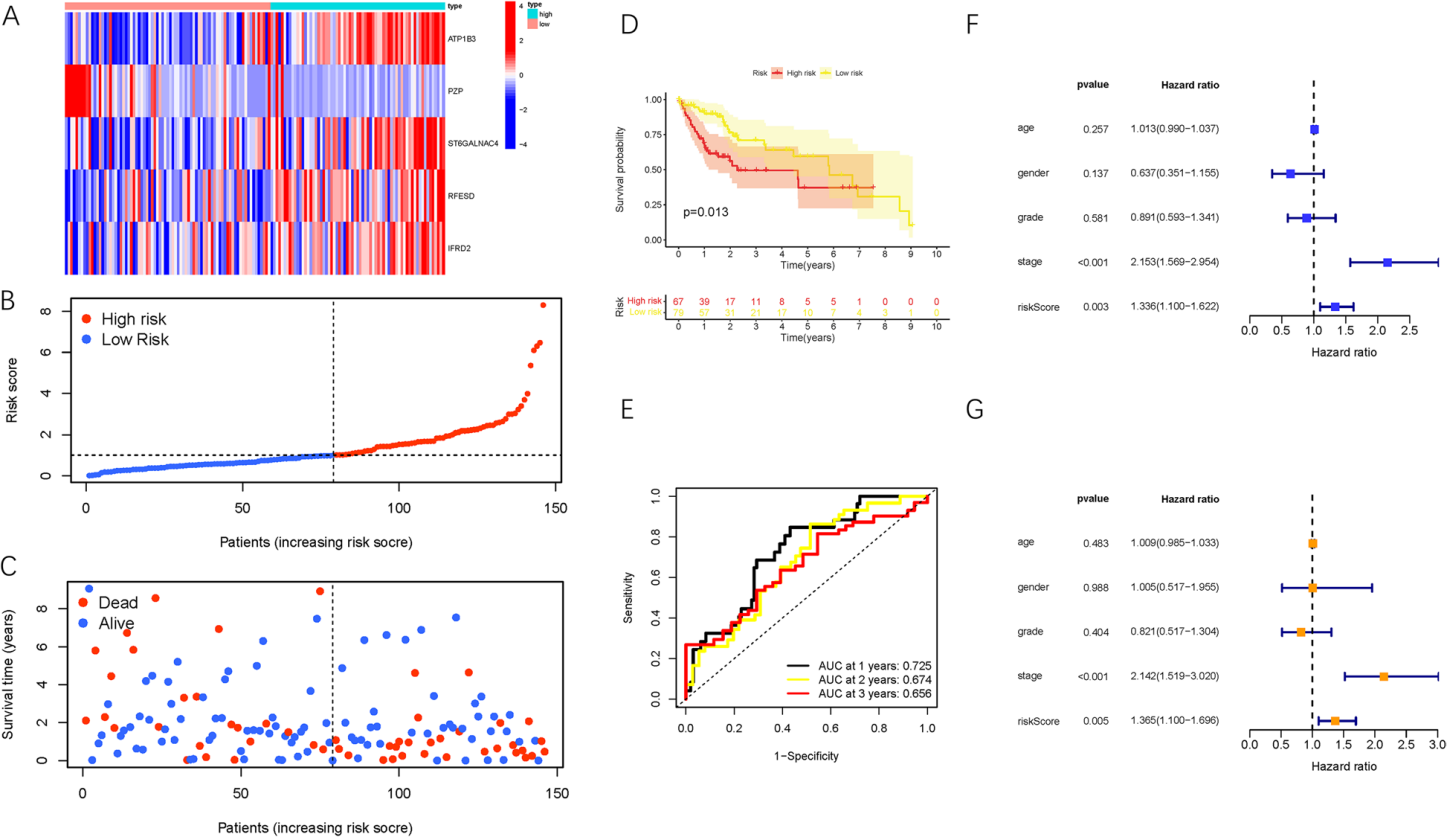
Confirmation of prognostic risk scores in the testing group. **A–E** presents testing cohort findings which are accordant with the training set results (Fig. 3). **F** Univariate Cox proportional hazards analyses of survival in the testing group. **G** Multivariate Cox proportional hazards analyses of survival in the testing group

These results were further validated in the internal testing group to demonstrate the prognostic performance of the constructed model. The distributions of gene expression patterns, survival status/time, and risk scores in the internal testing group and the entire HCC cohort are shown in Fig. 4A-C. Moreover, Kaplan-Meier curves were analyzed, and we found that low-risk HCC patients exhibited notably longer OS times compared with high-risk patients in the internal testing group (Fig. 4D, P =0.013). The areas under the ROC curves were both 0.74 or higher in the testing group (Fig. 4E), suggesting the robustness of this risk model for prognosis prediction in HCC. Likewise, risk score was as an independent prognosis prediction trait in both the univariable and multivariable regression analyses of the testing group (Fig. 4F-G).

The signature was applied to the ICGC-LIRI-JP cohort to validate the external prognosis prediction performance. Fig. S4A-C shows the distributions of the six gene expression patterns, sample survival status, and corresponding risk score in the external validation cohort. Furthermore, survival analysis showed that high-risk HCC patients had a poorer prognosis than low-risk patients (Fig. S4D, *p* = 0.003). The areas under the ROC values were more than 0.68 at 1, 2, and 3 years in the external testing cohort (Fig. S4E), which was consistent with our previous results in the training group. Taken together, our results confirm the external validity of the prognostic risk signature in distinct populations.

### Clinical significance of risk score

**Fig. 5:**
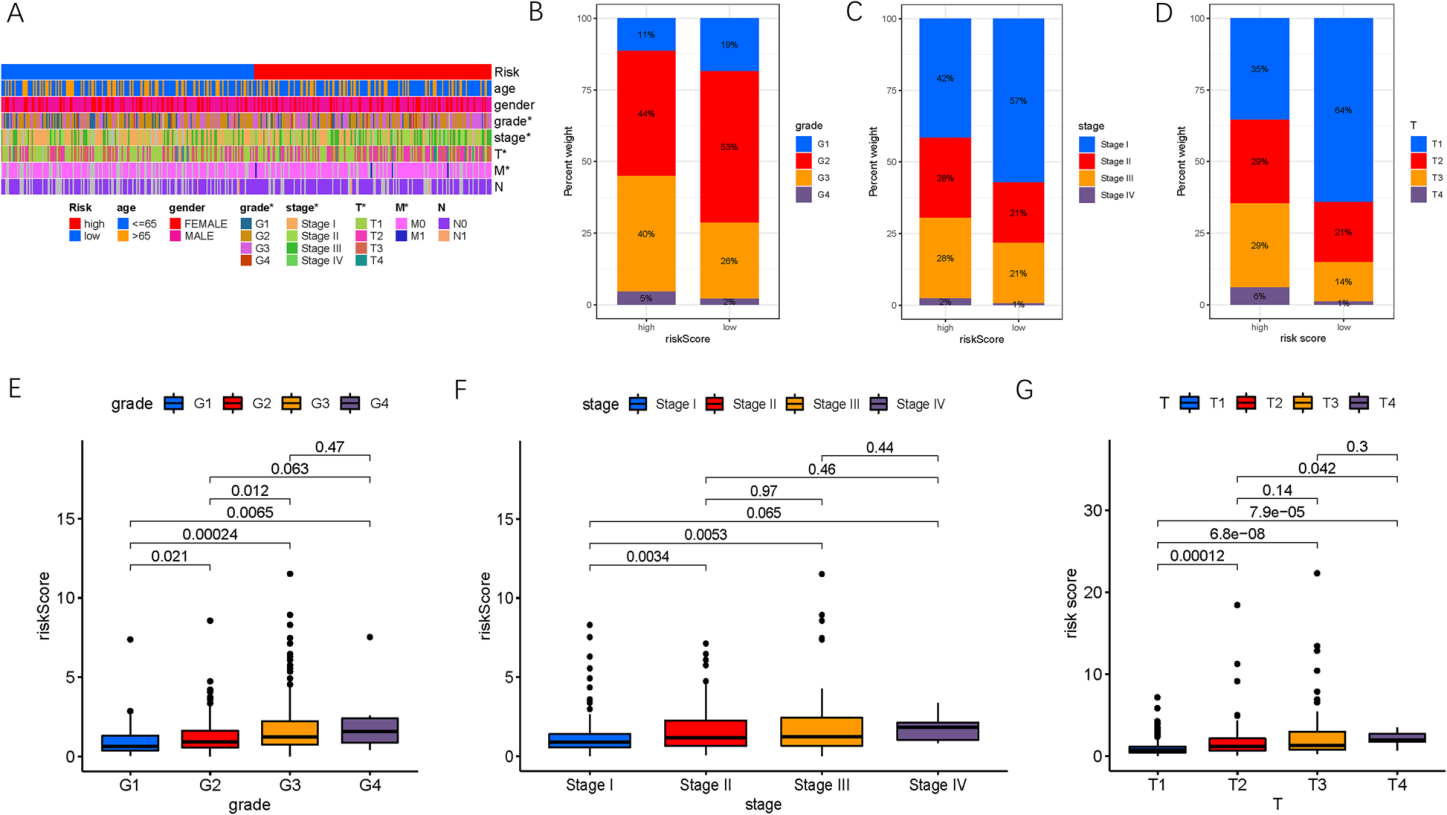
Clinical significance of the prognostic risk signature. **A** Heatmap presents the distribution of clinical feature and corresponding risk score in each sample. Rate of clinical variables subtypes in high or low risk score groups. **B** WHO grade, **C** clinical stage, and (**D**) T status. **E** Correlation of risk score with WHO grade. **F** Correlation of risk score with clinicopathological stage. **G** Correlation of risk score with T categories

Subsequently, the distribution of clinical variables in distinct risk subgroups was investigated (Fig. 5A). Figure 5B-D show the fraction of subgroups for clinicopathological grade, clinical staging, and T status in the low- and high-risk subgroups, respectively. We discovered that with advanced pathological grade (4/6, *P* < 0.05, Fig. 5E), late tumor staging (2/6, *P <* 0.05, Fig. 5F), and higher T status (4/6, *P <* 0.05, Fig. 5G), the risk score significantly increased.

Stratification survival analyses were performed to explore whether the risk score maintained its prognosis prediction capability when HCC patients were partitioned into subgroups with different clinical features. Relative to patients with high-risk HCC, samples from low-risk HCC patients showed higher OS probability in the ≤65- or > 65-year-old age subgroups (Fig. S5A, B). Similarly, the risk score showed great prognostic capability for both female and male patients (Fig. S5C, D), as well as tumor grades 1–2 or 3–4 (Fig. S5E, F), early- and late-stages (Fig. S5G, H), T1–2 or T3–4 categories (Fig. S5I, J), N0 status (Fig. S5K), and M0 category (Fig. S5L). These findings suggest that risk score may be an excellent prognostic predictor, independent of clinical traits.

### Correlation of risk with immune infiltration and therapeutic estimation

**Fig. 6:**
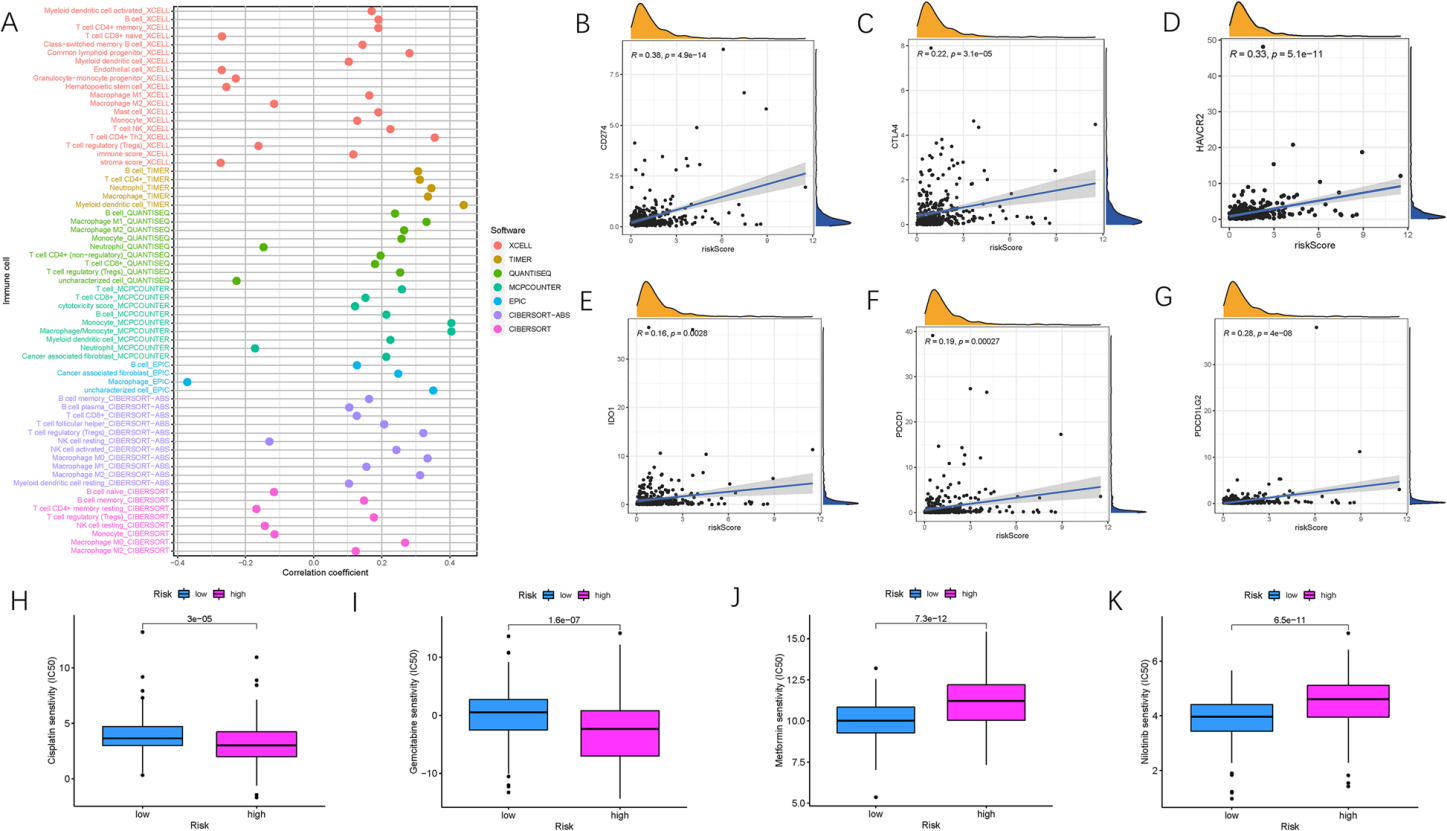
Estimation of Tumor-Infiltrating Cells and Immunotherapy significance. **A** Patients in the high-risk group were more positively associated with tumor-infiltrating immune cells such as macrophages, monocytes, and CD8+ T cells, whereas they were negatively associated with fibroblasts and CD4+ T cells, as shown by Spearman correlation analysis. Correlation between prognostic risk signature with hub immune checkpoint genes. **B** Correlation between prognostic risk signature and CD274, **C** Correlation between prognostic risk signature and CTLA4, **D** Correlation between prognostic risk signature and HAVCR2. **E** Correlation between prognostic risk signature and IDO1. **F** Correlation between prognostic risk signature and PDCD1. **G** Correlation between prognostic risk signature and PDCD1LG2. Estimation of Risk Score in Chemotherapeutic Effect. **H** Sensitivity analysis of Cisplatin in patients at high and low risk score. **I** Sensitivity analysis of Gemcitabine in patients at high and low risk score. **J** Sensitivity analysis of Metformin in patients at high and low risk score. **K** Sensitivity analysis of Nilotinib in patients at high and low risk score

As the γδ T cell-based risk signature was derived from immune infiltration profiling, the potential contribution of the risk score to immune infiltration was further explored. The results showed that risk score was significantly and negatively associated with infiltration of resting natural killer (NK) cells, whereas it was positively correlated with an abundance of CD8+ T cells, cancer-associated fibroblasts, monocytes, and M1 macrophages (Fig. 6A). The detailed results are presented in Table S8.

Subsequently, the possible roles of risk scores in immunotherapy were investigated. First, a correlation analysis of ICB hub targets (PDCD1, CD274, PDCD1LG2, CTLA-4, HAVCR2, and IDO1) [33–35] and risk score was conducted. The risk score was significantly and positively associated with the expression levels of CD274 (*r =* 0.38; *P* = 4.9e-14), CTLA-4 (*r =* 0.22; *P* = 3.1e-05), HAVCR2 (*r =* 0.33; *P* = 5.1e− 11), IDO1 (*r =* 0.16; *P* = 0.0028), PDCD1 (*r =* 0.19; *P* = 0.00027), and PDCD1LG2 (*r =* 0.28; P = 4e− 8; Fig. 6B-G), suggesting that risk score may act as a key driving factor in immunotherapeutic prediction of HCC.

Using the “pRRophetic” algorithm, the IC50 values of four chemotherapeutic drugs (cisplatin, gemcitabine, metformin, and nilotinib) were evaluated in HCC patients. Cisplatin and gemcitabine presented a lower IC50 trend in high-risk patients (both *p* < 0.05; Fig. 6H, I). Conversely, the IC50 of metformin and nilotinib was lower in HCC samples from low-risk patients (both *p <* 0.05; Fig. 6J, K). These findings demonstrate that patients from different risk subgroups were sensitive to distinct chemotherapeutic drugs.

### Enrichment of signaling pathways in low- and high-risk groups

**Fig. 7:**
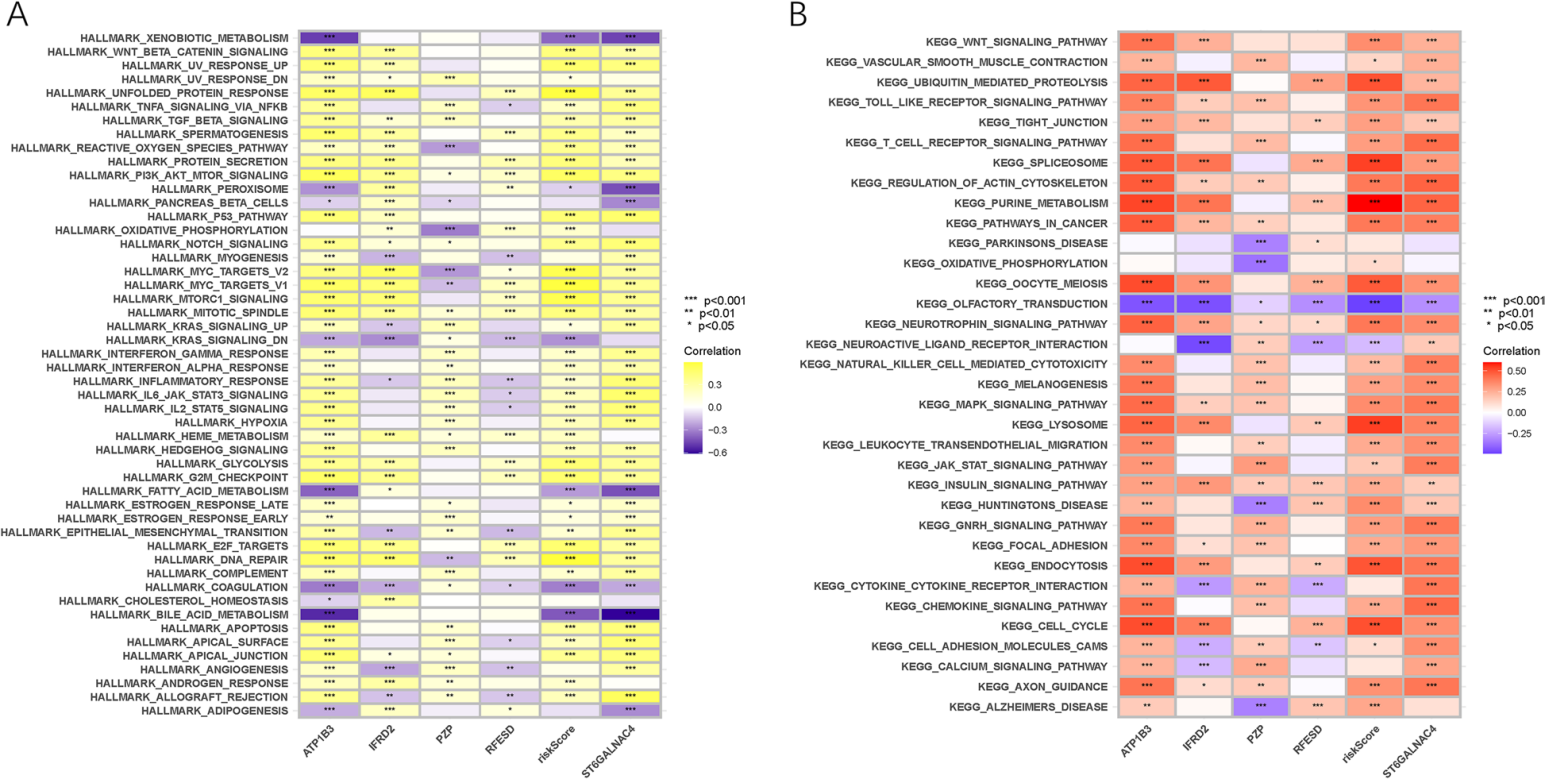
Enrichment pathways of GSVA. **A** Heatmap showing the correlation of representative pathway terms of Hallmark with risk score. **B** Heatmap showing the correlation of representative pathway terms of KEGG with risk score

To further reveal the biological roles of distinct risk groups in tumorigenicity and progression, GSVA was performed (Fig. 7A, B). The risk score was positively correlated with heightened activities of the Wnt-β-catenin, tumor necrosis factor (TNF)α/NF-κB, transforming growth factor (TGF)-β, phosphatidylinositol-3-kinase (PI3K)/AKT/mammalian target of rapamycin (Mtor), P53, Notch, interleukin (IL)6/Janus kinase (JAK)/signal transducer and activator of transcription (STAT)3, IL2/STAT5, mitogen activated protein kinases (MAPK), and KRAS signaling pathways. These results suggest that high-risk patients may be more suitable for administration of targeted molecular inhibitors. The risk score was negatively correlated with gene sets enriched in bile and fatty acid metabolism signaling pathways. To further reveal the underlying mechanism in low-risk patients, an investigation of metabonomic data is required in the future.

### Association between Risk and TMB.

**Fig. 8:**
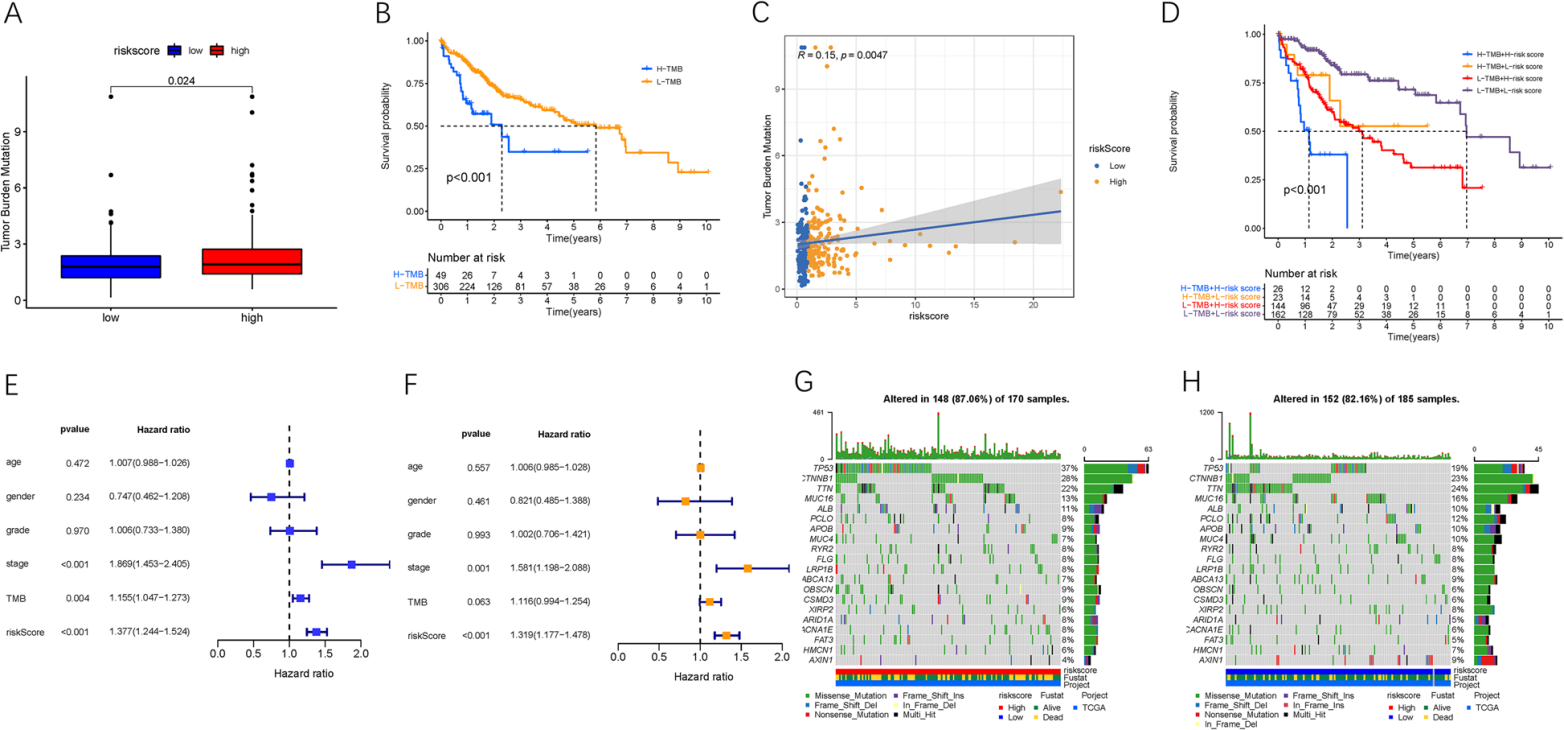
The Correlation between the risk Score and TMB. **A** Difference of TMB between patients from the low−/high-risk score subgroups. **B** Kaplan-Meier curves for high and low TMB groups. **C** Scatterplots depicting the positive correlation between risk scores and TMB. **D** Kaplan-Meier curves for patients stratified by both TMB and risk score. **E** Univariate Cox regression results of overall survival. **F** Multivariate Cox regression results of overall survival. The oncoPrint was constructed using high risk score (**G**) and low risk score (**H**)

Accumulating studies have provided strong evidence that TMB may act as a crucial indicator of sensitivity to anti-cancer treatment. Therefore, we investigated the intrinsic interaction of γδ T cell-based risk with TMB and elucidated the hereditary variations between risk subtypes. First, the TMB level of each HCC sample was measured. The TMB value showed a relatively lower trend in low-risk patients than in high-risk patients (*p* = 0.024, Fig. 8A). Subsequently, the HCC samples were divided into distinct subgroups based on the TMB value set point. Survival analysis further indicated that the TMB value can provide a significant prognostic difference (*p <* 0.001, Fig. 8B). Correlation analysis indicated that the TMB level was significantly and positively associated with risk score (*R =* 0.15, *p* = 0.0047, Fig. 8C). As revealed in the stratified survival curves, there was no interference of risk score with TMB value in prognosis prediction. The risk subgroups showed significant prognostic differences in both the low and high TMB value subtypes (*p <* 0.001, Fig. 8D). Furthermore, the multivariable regression analysis showed that TMB value was not an independent prognosis prediction factor for HCC (Fig. 8E, F). Our results suggest that risk score has the potential to predict anti-cancer therapy outcomes.

**Fig. 9:**
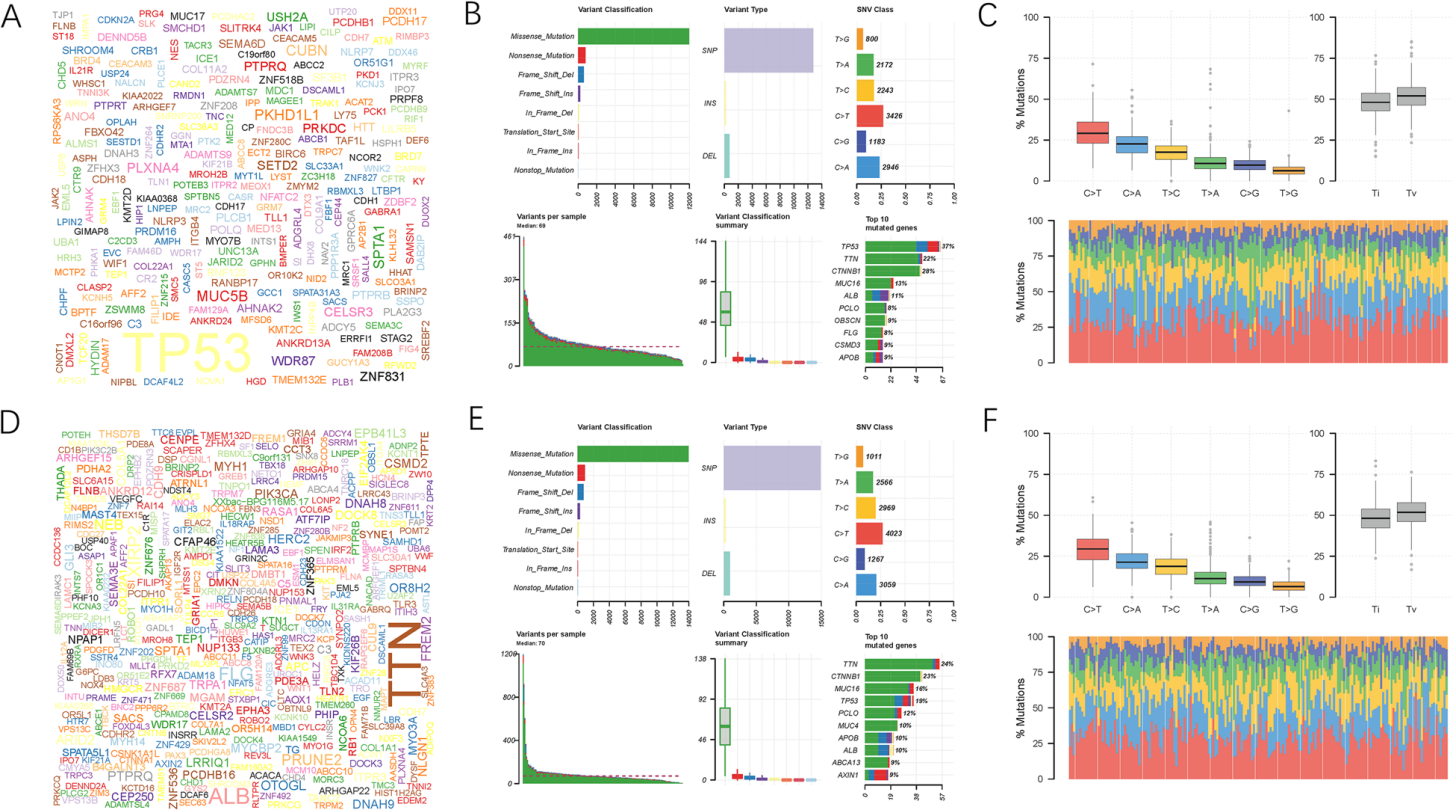
Landscape of somatic mutation profiles in low−/high-risk samples. **A** A word cloud generated based on frequency of mutated genes in low-risk subgroup. The size of each gene is proportional to the total number of samples mutated / altered. **B** Cohort summary plot of low-risk subgroup displaying distribution of variants according to variant classification, type and SNV class. Bottom part (from left to right) indicates mutation load for each sample, variant classification type. A stacked barplot shows top ten mutated genes. **C** Transition and transversion plot displaying distribution of SNVs in low-risk subgroup classified into six transition and transversion events. Stacked bar plot (bottom) shows distribution of mutation spectra for every sample in the MAF file. **D** A word cloud generated based on frequency of mutated genes in high-risk subgroup. The size of each gene is proportional to the total number of samples mutated / altered. **E** Cohort summary plot of high-risk subgroup displaying distribution of variants according to variant classification, type and SNV class. Bottom part (from left to right) indicates mutation load for each sample, variant classification type. A stacked barplot shows top ten mutated genes. **F** Transition and transversion plot displaying distribution of SNVs in high-risk subgroup classified into six transition and transversion events. Stacked bar plot (bottom) shows distribution of mutation spectra for every sample in the MAF file

Subsequently, the distribution of somatic variants was investigated in both the high- and low-risk subgroups. The comprehensive context of the gene mutations showed the variation patterns and clinical traits of the top 20 driver genes with the most frequent alterations (Fig. 8G, H). The mutational landscapes showed that TTN experienced the highest somatic mutation rates in the low-risk score subtype, whereas TP53 possessed the highest somatic mutation rates in the high-risk score subgroup (Fig. 9A, D). In the high-risk subgroup, missense mutation was the primary variant classification; most mutations belonged to single nucleotide polymorphisms, and C > T was the most common variation, with the highest number of variations per sample and the median of variation types (Fig. 9B, C). Similar results were observed in the low-risk subgroup (Fig. 9E, F). These results provide novel insights into the intrinsic interactions of somatic variants with γδ T cell patterns in HCC.

### Development of novel prognostic nomogram

**Fig. 10:**
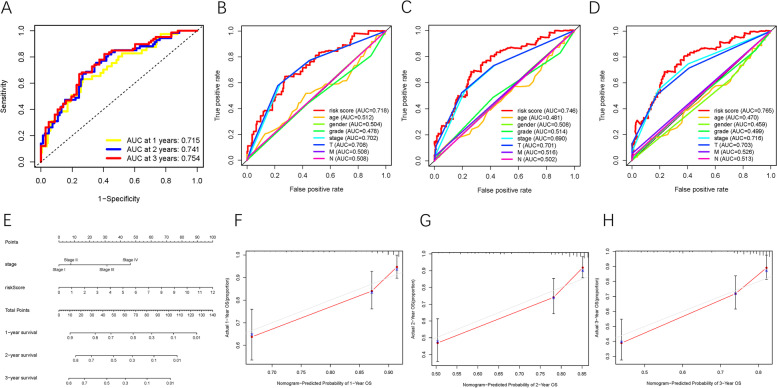
Validation of prognostic efficiency of risk signature in TCGA-LIHC. **A** ROC analysis was employed to estimate the prediction value of the prognostic signature. **B-D** Areas under curves (AUCs) of the risk scores for predicting 1-, 2-, and 3-year overall survival time with other clinical characteristics. **E** Nomogram was assembled by stage and risk signature for predicting survival of HCC patients. **F** One-year nomogram calibration curves. **G** Two-year nomogram calibration curves. **H** Three-year nomogram calibration curves

**Fig. 11:**
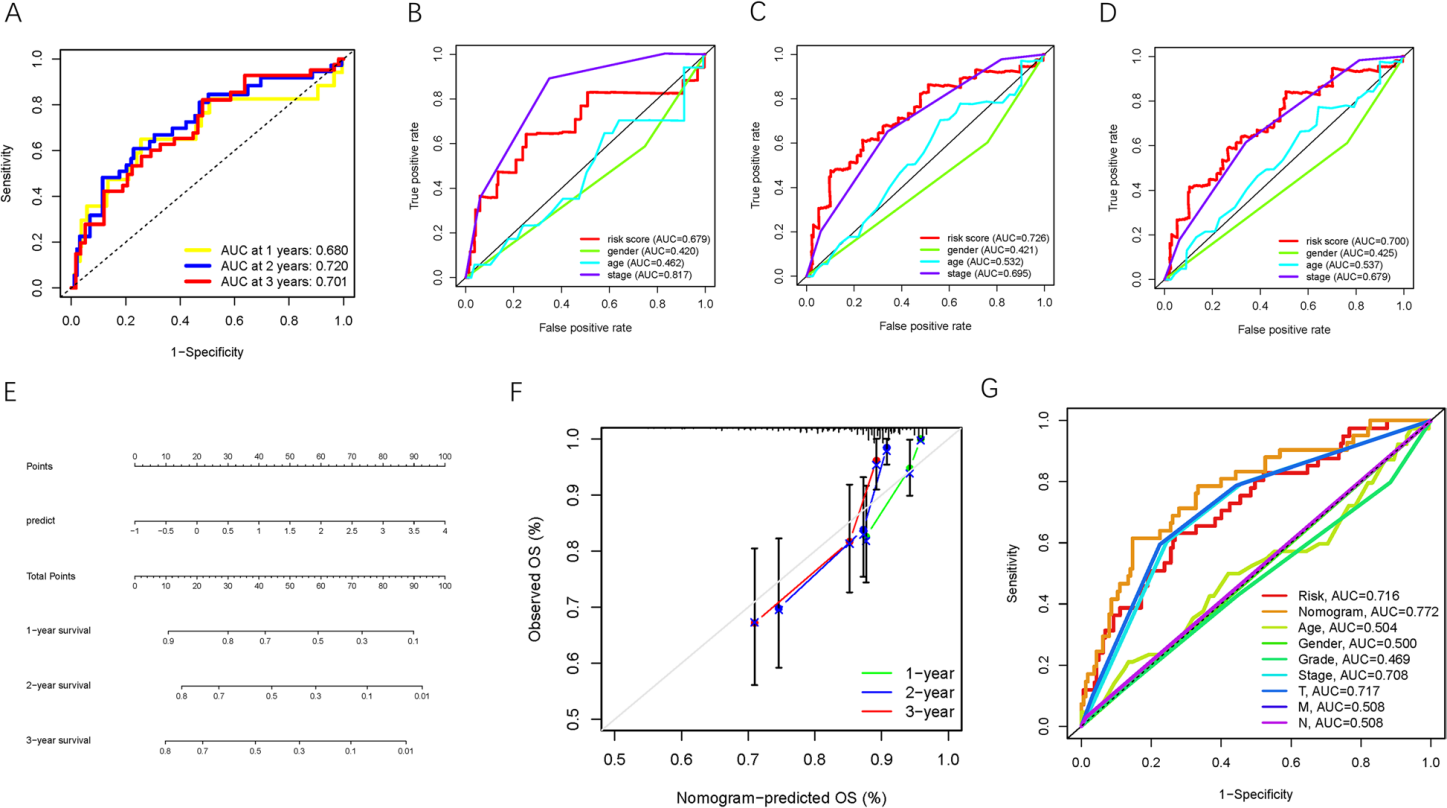
Testing of prognostic value of risk signature. **A** ROC analysis was employed to estimate the prediction value of the prognostic signature in the ICGC-LIRI-JP cohort. **B-D** Areas under curves (AUCs) of the risk scores for predicting 1-, 2-, and 3-year overall survival time with other clinical characteristics in the ICGC-LIRI-JP cohort. **E** Nomogram was assembled by stage and risk signature for predicting survival of HCC patients from ICGC-LIRI-JP cohort. **F** 1-, 2-, and 3-year nomogram calibration curves in the ICGC-LIRI-JP cohort. **G** ROC analysis was employed to estimate the prediction value of the prognostic nomogram in the TCGA-LIHC project

ROC curves were analyzed, and the AUC values for 1-, 2-, and 3-year OS were 0.715, 0.741, and 0.754, respectively, demonstrating the high predictive accuracy of the constructed risk model (Fig. 10A). To demonstrate that risk score was the best prognostic predictor among various clinical characteristics, age, sex, tumor stage, clinical grade, and TNM category were considered as candidate prognostic factors. These clinicopathological variables were used to implement the AUC analysis for 1-, 2-, and 3-year OS; risk score exhibited the highest AUC value (Fig. 10B-D). Subsequently, a novel nomogram, including risk score and clinical stage, was established for OS probability prediction (Fig. 10E). Age, sex, and tumor grade were excluded from the nomogram because their AUC values were less than 0.6. Calibration curves were plotted, which suggested high prognostic accuracy of the risk-stage nomogram (Fig. 10F-H). In addition, this nomogram was used in the ICGC-LIRI-JP cohort to confirm its external prognostic ability. Figure 11A shows the AUC values for 1-, 2-, and 3-year OS of the risk scores in the external validation cohort. Moreover, risk and stage were good indicators with AUC values > 0.6 (Fig. 11B-D). The survival rates of HCC patients from the ICGC-LIRI-JP cohort were predicted using the constructed nomogram (Fig. 11E) and its prognostic robustness was validated (Fig. 11F). Finally, the ROC curve was analyzed to confirm the prognostic performance of the nomogram relative to other prognostic factors in TCGA-LIHC project (Fig. 11G).

### Potential function of RFESD in prognosis prediction, mechanism of immune infiltration, and therapeutic estimation

**Fig. 12:**
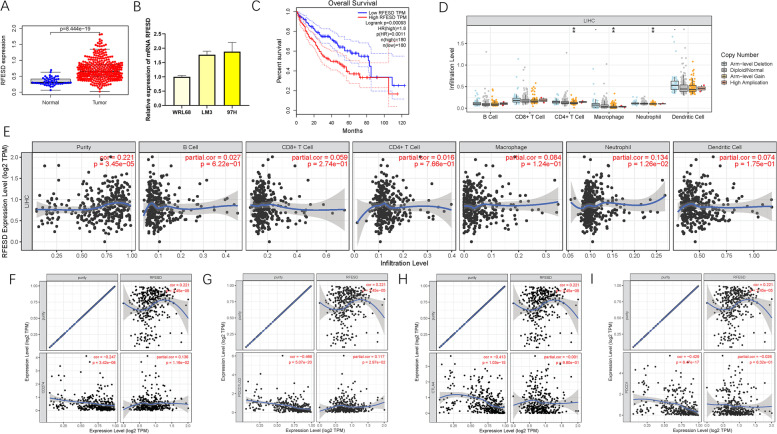
The clinical significance of RFESD in HCC. FAM53B are upregulated in HCC samples based on TCGA dataset**A** and cell lines (**B**), and lower RFESD expression level was significantly correlated with improved prognosis (**C**). (**D**) Copy number of immune cells in HCC. **E** Correlation analysis of RFESD with infiltrating B cells, CD4 + T cells, CD8 + T cells, Macrophages, Neutrophils and Dendritic cells using TIMER. The association between the expression levels of RFESD with CD274 (**F**), PDCD1LG2 (**G**), CTLA4 (**H**), and PDCD1 (**I**) using TIMER

The potential role of RFESD as a prognostic γδ T cell-related gene has not yet been revealed yet in HCC. Thus, the biological roles of RFESD were further explored in subsequent analyses. The expression values of RFESD were compared between normal and tumor tissue samples based on TCGA dataset. For normal specimens and tumor tissues, RFESD expression levels showed a lower trend in normal compared with tumor tissue samples (Fig. 12A). Using qRT-PCR, the expression levels of RFESD in hepatic cell lines and two distinct hepatic cancer cell lines were investigated. Likewise, hepatic cancer cells exhibited significantly higher RFESD values than normal liver cells (Fig. 12B). To estimate the prognosis prediction performance of RFESD, survival analysis was performed to compare HCC patients with low and high RFESD expression. These results showed that a higher expression value of RFESD was significantly associated with a shorter OS time (*P* = 0.00093, Fig. 12C).

To reveal the biological roles of RFESD in immune infiltration, the correlation between RFESD expression and the abundance of infiltrating immune cells was investigated using the TIMER database. Arm-level gain was the main mutation type in CD4+ T cells, macrophages, and neutrophils (Fig. 12D). In addition, the expression level of RFESD was significantly correlated with neutrophil infiltration (*r =* 0.134; *P* = 1.26e− 02; Fig. 12E).

Next, the correlation of RFESD with key ICB genes adjusted by tumor purity was analyzed to investigate the potential functions of RFESD in immunotherapeutic prediction. These results showed that RFESD had a significant positive association with two of the four key ICB genes, including CD274 (*r =* 0.136; *P* = 1.16e− 02) and PDCD1LG2 (*r =* 0.117; *P* = 2.97e− 02; Fig. 12F-I), indicating that RFESD is an indispensable regulator in HCC immunotherapy.

### Downregulation of RFESD suppressed HCC cell proliferation

**Fig. 13:**
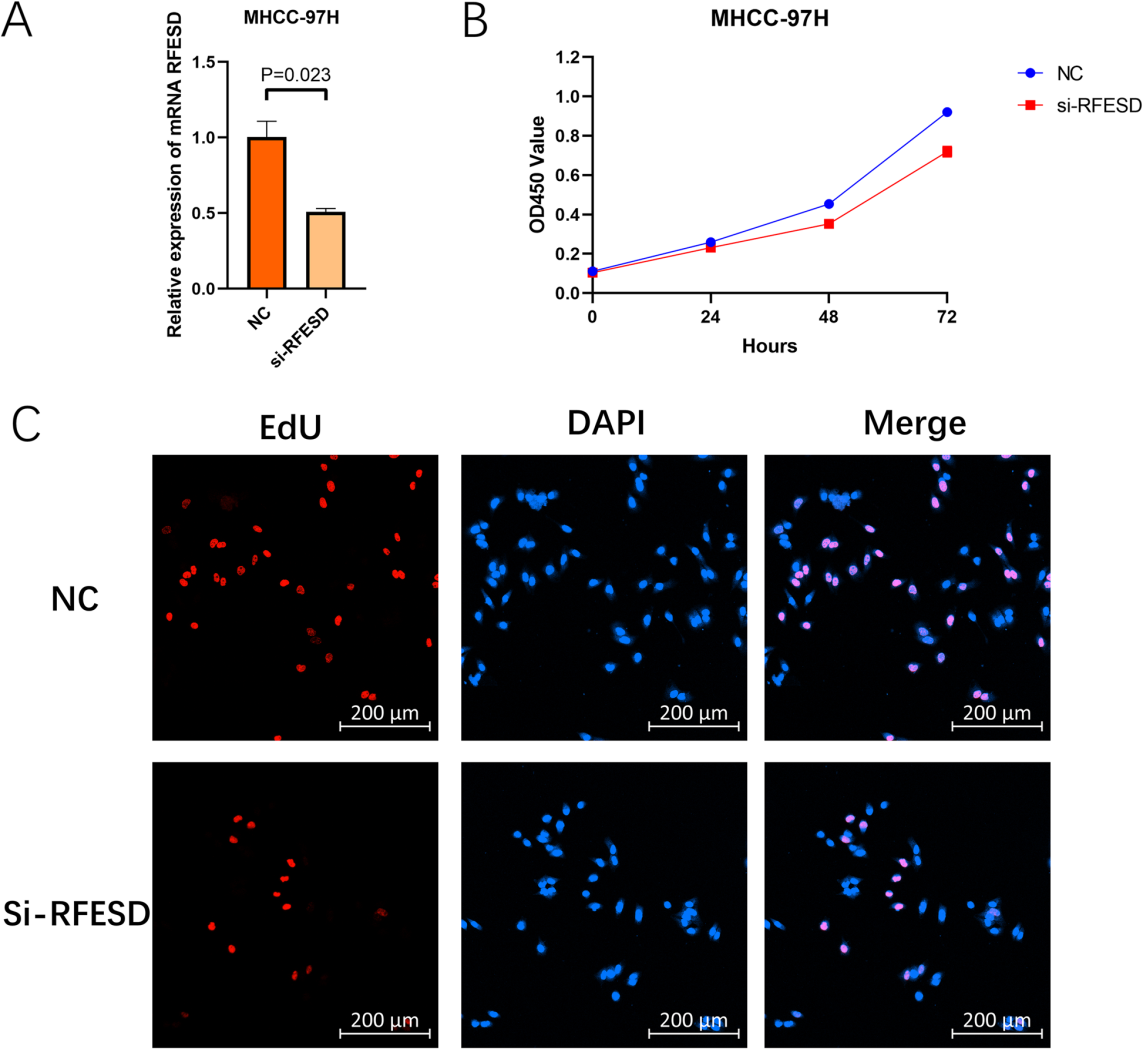
The clinical significance of RFESD in HCC and in vitro study. **A** Transfection efficiency was verified after transfection of RFESD or negative control siRNA. **B** HCC cell viability was evaluated with CCK-8 assays at 0, 24, 48, and 72 h post-transfection. **C** The growth of HCC cells was estimated by EdU assays after RFESD knockdown

To further reveal the biological roles of RFESD, RFESD siRNA was used to silence RFESD protein expression. The transfection effect on HCC cells was first estimated by qRT-PCR, and we observed that the relative mRNA expression value of RFESD was significantly lower after transfection with siRNA (Fig. 13A, P = 0.023). To further reveal the possible role of RFESD in cell proliferation, CCK-8 assays were performed to evaluate the effect of RFESD silencing on HCC cell growth. After RFESD knockdown, MHCC-97H cell proliferation was significantly decreased relative to that in control cells (Fig. 13B, P < 0.05). The EdU assay results also suggested that RFESD silencing significantly repressed HCC cell proliferation (Fig. 13C). These findings highlight that RFESD knockdown inhibits the proliferative abilities of HCC cells.

## Discussion

HCC is one of the most prevalent malignant tumors and is characterized by increased mortality worldwide [1–3]. It is well acknowledged that alternative splicing, TP53 mutation and regulation of non-coding RNA and DNA methylation play crucial roles in HCC progression [5, 35–40]. Recently, an increasing number of studies have provided evidence for the non-negligible functions of immune infiltration in the progression of cancer, including HCC [13, 41–43]. Immunotherapy has been designed to harness immune cells to annihilate cancer cells and has exerted encouraging therapeutic effects and promising clinical outcomes in anticancer therapy [44]. The results of previous clinical studies suggest that the administration of ICB in advanced HCC patients has observable benefits; however, only 20% of patients had an objective response to treatment (57). Biomarkers such as ICB-related hub targets are unreliable for therapeutic prediction. Therefore, it is imperative to predict clinical outcomes to optimize therapeutic benefits and individualize treatment plans [31, 45, 46].

Upon review of the articles, we found that an increasing number of studies have focused on immune infiltration in human HCC [47, 48], especially γδ T cells [49]. A previous study showed that incubation of γδ T cells with hepatic tumor cell lines resulted in a significant decrease in cancer cell viability [50]. Xi et al.. reported HP1 and MSP as potential candidates for antigens recognized by γδ T cells in HCC [51]. It is well established that γδ T cells act as a bridge between the adaptive and innate immune systems and mediate various immune responses during tumor development [52]. Besides, γδ T cells with features of abundant cytokine secretion capacity and non-major histocompatibility complex restricted antigen recognition have attracted interest for their application in malignant tumor immunotherapy [53]. These results emphasize that γδ T cell patterns may play a central role in tumor progression and response to clinical treatment.

Herein, we gathered three distinct HCC cohorts from the GSE54236, TCGA-LIHC, and ICGC-LICA-FR datasets to investigate the possible functions of γδ T cell-specific genes in distinct populations. In total, 616 tumor tissues, 130 normal samples, and 16,421 corresponding genes were used in the subsequent analyses. First, the CIBERSORT algorithm was employed to estimate the relative abundance of 22 immune infiltrates. The most significant modules (brown) with 1755 candidate genes were identified and labelled γδ T cell-specific genes using the WGCNA algorithm. In addition, the results of functional annotation showed that these key genes were predominantly enriched in immunological activity, especially T-cell activation. Moreover, we discovered that abnormal expression levels of these hub genes significantly affected the OS time in HCC patients.

To further demonstrate the prognostic validity of these hub genes, sequencing profiling and clinical data were obtained from TCGA-LIHC project. Univariate, LASSO, and multivariate COX analyses were conducted to determine the final five hub genes, the risk score was computed, and a prognostic risk signature was constructed. The prognostic value of the as-constructed risk model was demonstrated using Kaplan-Meier analysis and ROC curves. The risk signature was found to perform well as an independent prognosis prediction factor in both the univariate and multivariate regression analyses. Further validation was performed using an external dataset (ICGC-LIRI-JP cohort). Subsequently, we observed that the risk score was positively correlated with the clinical grade, tumor stage, and T category. In addition, the risk signature still held powerful prognostic capability in the clinical feature-stratified survival analyses. A novel risk stage nomogram was established for further clinical practice. Finally, the prognosis prediction performance of our nomogram was validated in an external testing group.

Given the risk signature derived from immune infiltrating status, the potential role of the risk score in the mechanism of immune infiltration and therapeutic evaluation were further investigated. The results showed that risk score was negatively correlated with infiltration of dysfunctional immune cells (i.e., resting NK cells), whereas it was positively correlated with the abundance of activated immune cells (i.e., CD8+ T cells), highlighting that tumors with a high-risk score could be termed the immune-activated phenotype. Additionally, the risk score was positively and significantly correlated with the expression levels of key ICB targets (i.e., CD274), indicating that high-risk HCC samples might be more affected by ICB pathways, thereby inhibiting anti-tumor immune activation and deteriorating prognosis accordingly. Because there were no immunotherapy data for the HCC cohort, we were unable to further investigate the potential correlation between risk score and immunotherapeutic response.

It is worth mentioning that the GSVA results indicated that bile acid and fatty acid metabolism signaling pathways were activated in the low-risk group, whereas the Wnt-β-catenin, TNFα/ NF-κB, TGF-β, PI3K/AKT/mTOR, P53, Notch, IL6/JAK/STAT3, IL2/STAT5, MAPK, and KRAS signaling pathways were activated in the high-risk group. These results showed that the underlying molecular mechanisms varied significantly between different risk samples. In addition, the risk-scoring scheme revealed that the sensitivity of chemotherapy drugs was associated with risk score. Therefore, HCC patients might be more suitable for a distinct combination of targeted molecular therapy and chemotherapeutic agents according to risk stratification.

Several recent clinical studies have reported a correlation between genetic alterations and immunotherapeutic sensitivity [54, 55]. Our results showed that TMB, a predictive indicator of immunotherapeutic responsiveness, increased significantly as the risk score increased. Subsequent stratified survival analyses supported that risk scores remain a powerful prognosis predictor independent of TMB values, indicating that TMB level and risk score represent distinct aspects of immunobiology in HCC. Moreover, risk score together with mutation information revealed significant differences in gene variant frequency between the low- and high-risk subgroups. Interestingly, the low-risk subgroup shared a similar SNPs status to the high-risk subgroup.

According to the reviewed articles, studies focusing on the biological functions of RFESD in cancer have not yet been published. Herein, its prognosis prediction performance and effects on TIME context and cell growth were explored. RFESD was significantly overexpressed in HCC cell lines and served as a poor prognosis prediction indicator of HCC. Additionally, RFESD exhibited a notable correlation with the infiltration of immune cells (i.e., neutrophils) in HCC. Additionally, RFESD may play a role in promoting the proliferation of HCC cell lines. Furthermore, the expression of RFESD was significantly positively correlated with immunotherapeutic hub genes (i.e., CD274, PDCD1LG2). However, the potential molecular mechanisms of RFESD in HCC progression remain elusive and require further experimental validation.

## Conclusions

In summary, the comprehensive landscape of TIME was delineated using distinct datasets and multiple bioinformatics analyses. Additionally, the distinction of the γδ T cell patterns was found to contribute to differences in clinical outcomes and TIME feature heterogeneity. The potential mechanism pathways and chemotherapeutic drugs were investigated under different risks. In addition, the synergistic effect of the risk score and TMB value was demonstrated in the prognosis prediction. To our knowledge, this is the first study to investigate the biological role of RFESD in HCC. Finally, a novel and robust nomogram was developed for quantitative estimation of patient risk. However, subsequent experimental and clinical validation at different centers with larger cohorts are required to validate our results.

## Supplementary Information

**Additional file 1.**

**Additional file 2.**

## Data Availability

The datasets generated for this study can be found in the TCGA database (https://portal.gdc.cancer.gov), and GEO database (https://www.ncbi.nlm.nih.gov/geo/). The original data was too large to upload in the system, so they were uploaded in the Google Drive. The download link was as follow: https://www.jianguoyun.com/p/Df1lb_0Q_fScChi_yLYEIAA

## Abbreviations

AUC: Area under the curve
BP: Biological processes
CC: Cellular components
CD274: Also known as PD-L1
CI: Confidence Interval
CTLA-4: cytotoxic T-lymphocyte antigen 4
CTLs: Cytotoxic T lymphocytes
DCs: Dendritic cells
DEG: Differentially expressed genes
DEL: Deletion
FDR: False discovery rate
GO: Gene ontology
γδT: Gamma delta T
GSEA: Gene set enrichment analysis
HCC: Hepatocellular carcinoma
HR: Hazard ratio
ICB: Immune checkpoint blockade
IDO1: Indoleamine 2,3-dioxygenase 1
KEGG: Kyoto Encyclopedia of Genes and Genomes
K-M: Kaplan-Meier
K-W: Kruskal-Wallis
LASSO: Least absolute shrinkage and selection operator
MF: Molecular function
OS: Overall survival
PDCD1: Also known as PD-1
PDCD1LG2: Also known as PD-L2
ROC: Receiver operating characteristic
TCGA: The Cancer Genome Atlas
TICs: Tumor-infiltrating immune cells
TILs: Tumor Infiltrating Lymphocytes
TIM-3: T-cell immunoglobulin domain and mucin domain-containing molecule-3
TIME: Tumor immune microenvironment
TMB: Tumor mutation burden
TNM: Tumor Node Metastasis
Tregs: Regulatory T cells
WGCNA: Weighted gene co-expression network analysis
