# Supplementary material for: Novel γδ T cell-based prognostic signature to estimate risk and aid therapy in hepatocellular carcinoma
Source: BMC Cancer. 2022 Jun 10;22:638. doi: 10.1186/s12885-022-09662-6 (PMC9185956; doi:10.1186/s12885-022-09662-6)
Supplement: Supplementary file 2 — Additional file 2. [file 12885_2022_9662_MOESM2_ESM.pdf]

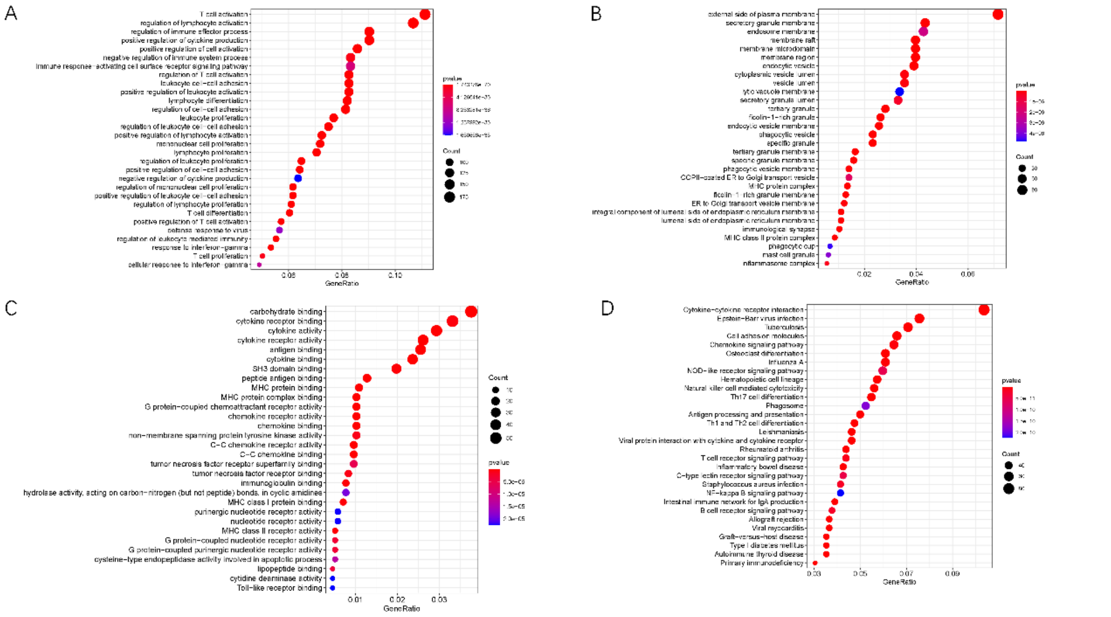

Figure S1: Pathway enrichment analyses of γδT cells-related genes. Gene Ontology (GO) enrichment analysis of γδT cells-related genes: biological processes (BP) (A), cellular components (B) and molecular function (C). (D) KEGG enrichment analysis of γδT cells-related genes.

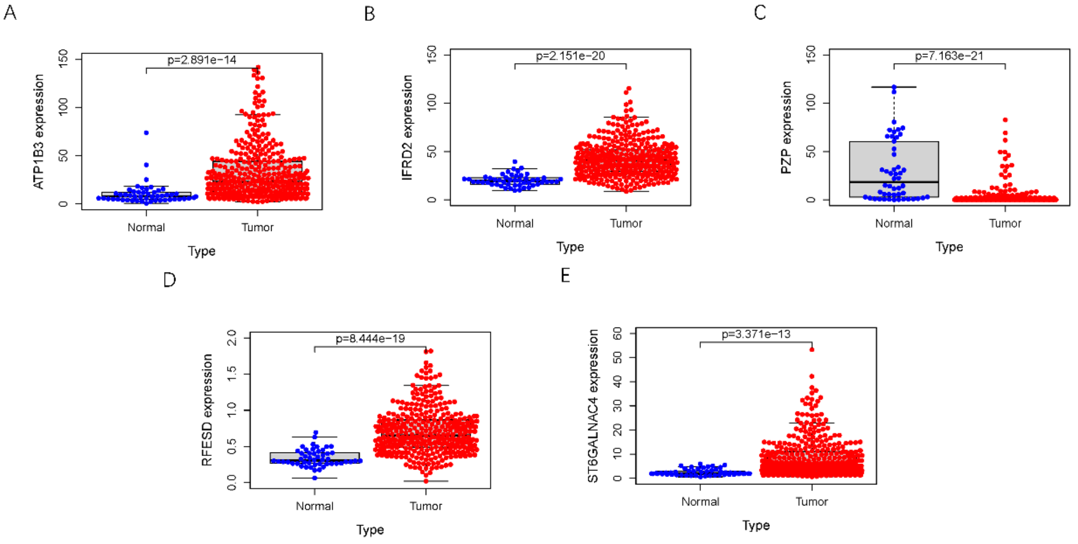

Figure S2: The mRNA expression level of hub genes in TCGA cohort. (A) ATP1B3, (B) IFRD2, (C) PZP, (D) RFESD, (E) ST6GALNAC4

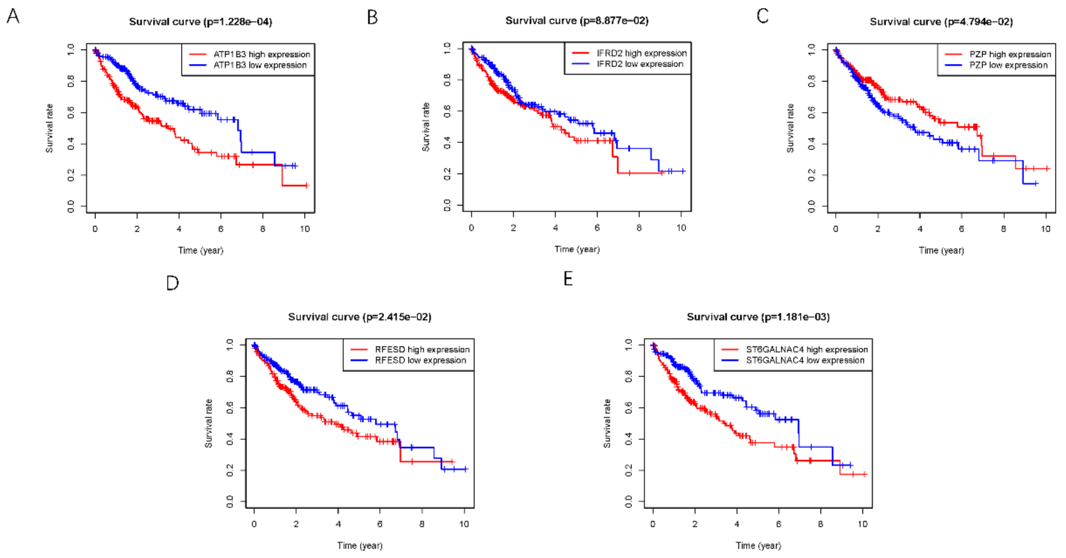

Figure S3: Survival analysis between high- and low- expression groups of hub genes. (A) ATP1B3, (B) IFRD2, (C) PZP, (D) RFESD, (E) ST6GALNAC4

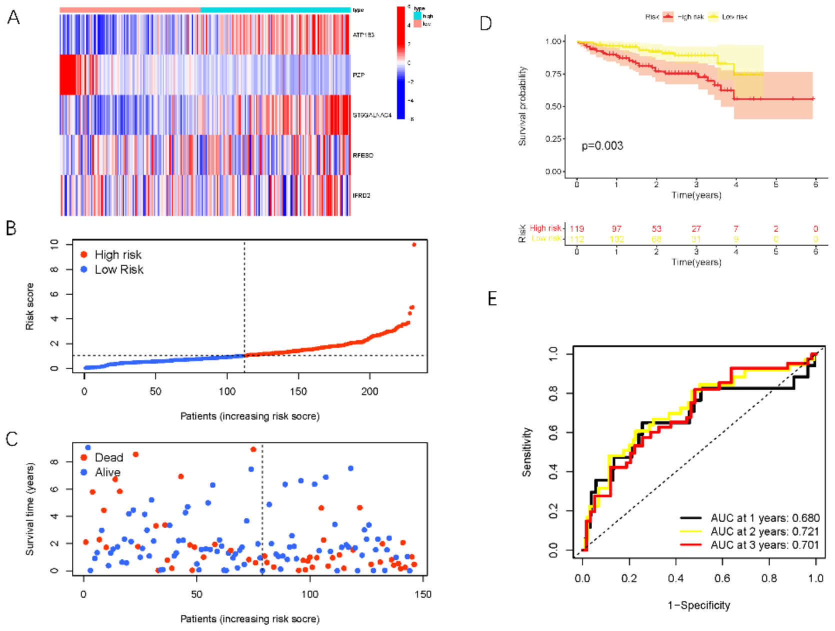

Figure S4. Validation of the prognostic value of risk signature. (A) Heatmap presents the expression pattern of three hub genes in each patient, where the colors of yellow to blue represented alterations from high expression to low expression. (B) Distribution of multi-genes signature risk score. (C) The survival status and interval of HCC patients. (D) Kaplan–Meier curve analysis presenting difference of overall survival between the high-risk and low-risk groups. (E) Areas under curves (AUCs) of the risk scores for predicting 1-, 2-, and 3-year overall survival time.

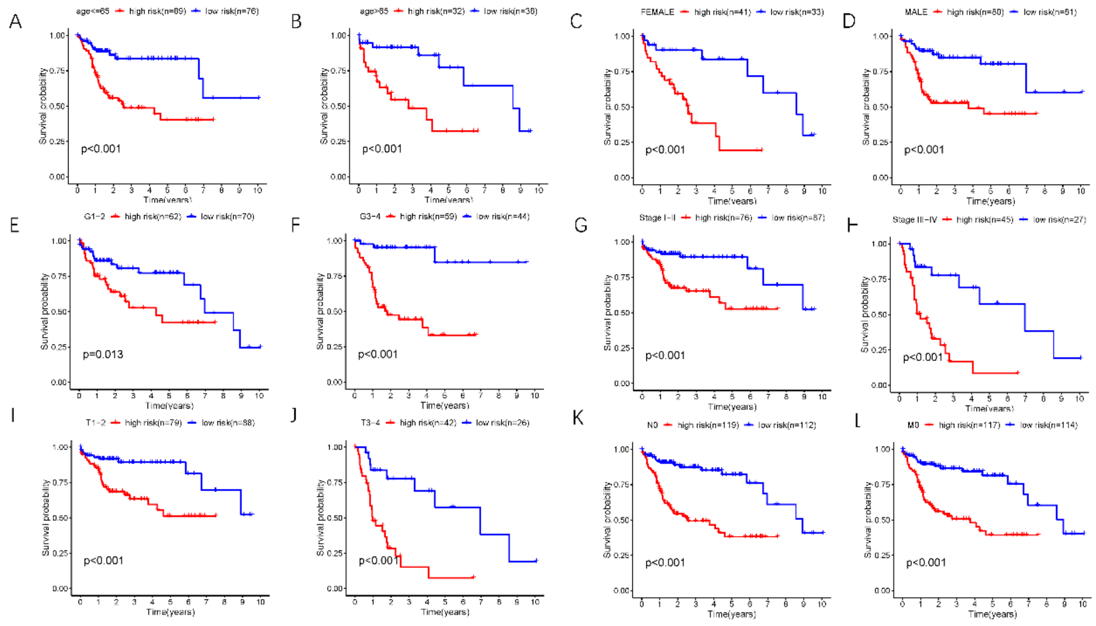

Figure S5: Kaplan–Meier survival analysis for multiple HCC subgroups according to the risk signature stratified by clinical variables. (A-B) Age. (C-D) Gender. (E-F) Tumor grade. (G-H) Stage. (I-J) T status. (K) N status. (L) M status.
